# Peroxisome deficiency but not the defect in ether lipid synthesis causes activation of the innate immune system and axonal loss in the central nervous system

**DOI:** 10.1186/1742-2094-9-61

**Published:** 2012-03-29

**Authors:** Astrid Bottelbergs, Simon Verheijden, Paul P Van Veldhoven, Wilhelm Just, Rita Devos, Myriam Baes

**Affiliations:** 1 Laboratory of Cell Metabolism, Department of Pharmaceutical Sciences, K.U.Leuven, Leuven, Belgium; 2 LIPIT, Department of Molecular Cell Biology, K.U. Leuven, Leuven, Belgium; 3 Heidelberg Center of Biochemistry, University of Heidelberg, Heidelberg, Germany; 4 Department of Pathology, K.U.Leuven, Leuven, Belgium

**Keywords:** Peroxisomes, Mouse models, Plasmalogens, Complement, Demyelination, Axonal degeneration, Inflammation, Macrophage

## Abstract

**Background:**

Mice with peroxisome deficiency in neural cells (*Nestin-Pex5*^*−/−*^*)* develop a neurodegenerative phenotype leading to motor and cognitive disabilities and early death. Major pathologies at the end stage of disease include severe demyelination, axonal degeneration and neuroinflammation. We now investigated the onset and progression of these pathological processes, and their potential interrelationship. In addition, the putative role of oxidative stress, the impact of plasmalogen depletion on the neurodegenerative phenotype, and the consequences of peroxisome elimination in the postnatal period were studied.

**Methods:**

Immunohistochemistry in association with gene expression analysis was performed on *Nestin-Pex5*^*−/−*^ mice to document demyelination, axonal damage and neuroinflammation. Also *Gnpat*^*−/−*^ mice, with selective plasmalogen deficiency and *CMV-Tx-Pex5*^*−/−*^ mice, with tamoxifen induced generalized loss of peroxisomes were analysed.

**Results:**

Activation of the innate immune system is a very early event in the pathological process in *Nestin-Pex5*^*−/−*^ mice which evolves in chronic neuroinflammation. The complement factor C1q, one of the earliest up regulated transcripts, was expressed on neurons and oligodendrocytes but not on microglia. Transcripts of other pro- and anti-inflammatory genes and markers of phagocytotic activity were already significantly induced before detecting pathologies with immunofluorescent staining. Demyelination, macrophage activity and axonal loss co-occurred throughout the brain. As in patients with mild peroxisome biogenesis disorders who develop regressive changes, demyelination in cerebellum and brain stem preceded major myelin loss in corpus callosum of both *Nestin-Pex5*^*−/−*^ and *CMV-Tx-Pex5*^*−/−*^ mice. These lesions were not accompanied by generalized oxidative stress throughout the brain. Although *Gnpat*^*−/−*^ mice displayed dysmyelination and Purkinje cell axon damage in cerebellum, confirming previous observations, no signs of inflammation or demyelination aggravating with age were observed.

**Conclusions:**

Peroxisome inactivity triggers a fast neuroinflammatory reaction, which is not solely due to the depletion of plasmalogens. In association with myelin abnormalities this causes axon damage and loss.

## Background

Patients with peroxisomal dysfunction present with severe and diverse neurological anomalies, including neuronal migration defects, dysmyelination and inflammatory demyelination and axon damage, proving that these organelles are indispensible for the normal development and maintenance of the central nervous system (CNS) [1-3]. According to the genetic causes these diseases can be categorized in peroxisome biogenesis defects (PBDs) and in single enzyme or transporter deficiencies. The PBDs are due to a mutation in a *PEX* gene, encoding a peroxin involved in the assembly of the organelles. The enzyme/transporter defects mostly involve 1) the peroxisomal α-oxidation pathway, necessary for the breakdown of phytanic acid and long chain 2-hydroxy fatty acids, 2) β-oxidation, which is required for degradation of very long chain fatty acids and pristanic acid, as well as the synthesis of polyunsaturated fatty acids and bile acids and 3) ether phospholipid synthesis which include plasmalogens.

To investigate the postnatal pathologies in the CNS, a mouse model with neural selective peroxisome dysfunction was generated by breeding *Nestin-Cre* mice with *Pex5-loxP* mice. In the latter mice the gene encoding the import receptor of peroxisomal matrix proteins is floxed [4]. This model shows a mild and temporary delay in neurodevelopment [5] but from 3 weeks on *Nestin-Pex5* knockout mice display motor and later on cognitive abnormalities, aggravating with increasing age and evolving in immobility and death before the age of 6 months. In brain, severe dys- and demyelination, astro- and microgliosis and axonal damage were observed [4]. However, the relationship between these anomalies and the precise onset and progression of pathologies in different brain areas were not elucidated. It was further demonstrated that a similar but less aggressive phenotype develops in mice with oligodendrocyte selective inactivation of *Pex5*[6], whereas mice with neuron or astrocyte selective deletion of functional peroxisomes were spared from demyelination, axon damage and astro- and microgliosis [7].

*Gnpat*^*−/−*^ mice [8], which lack a crucial enzyme of ether lipid synthesis, also exhibit a brain phenotype. The cerebellar fibers display hypomyelination at the age of 3 weeks which does not aggravate at 6 weeks, based on microscopical investigations and western blot analysis. Significant dysmyelination was also observed in the outer neocortical fibers of juvenile and adult (aged 8 months) *Gnpat*^−/−^ mice. Furthermore, they display disturbed axoglial contacts resulting in abnormal paranodal organisation and axonal swellings [9]. In lipid raft microdomains (LRMs) isolated from myelin a significant reduction of LRM proteins was found, which was ascribed to the severe lack of plasmalogens [8].

Also impaired peroxisomal β-oxidation causes a postnatal phenotype in the CNS. *Abcd1* knockout mice, a model for the adrenomyeloneuropathy (AMN) form of X-linked adrenoleukodystrophy (X-ALD), cannot transport a subset of substrates over the peroxisomal membrane, presumably saturated and/or unsaturated very long chain fatty acid CoA esters. They develop a late onset axonopathy in the spinal cord, but no brain defects. Recently, oxidative stress was detected in spinal cord of X-ALD mice, long before these mice develop motor abnormalities [10]. Treatment with anti-oxidants inhibited the development of oxidative stress and prevented the development of motor disability and axonal damage [11]. Furthermore, in brain-selective *Pex13* knockout mice [12] increased superoxides and MnSOD were detected in cerebellar cell cultures and up regulation of MnSOD in the Purkinje cell layer of the cerebellum in vivo.

Mice deficient in MFP-2 (also denoted as D-bifunctional protein), carry a broader defect in peroxisomal β-oxidation, as this enzyme is necessary for the degradation of both straight and branched chain substrates [13]. They bear several similarities with *Nestin-Pex5* knockout mice in view of their motor defects and early death. Marked astro- and microgliosis were described [14] but no thorough study of myelinated axons was performed.

Although we already reported on the pathology in the end phase of disease of mice with peroxisome deficiency in brain, the aim of the present investigation was to better define the onset and progression of pathological events in different brain areas of *Nestin-Pex5*^*−/−*^ mice. Therefore, immunofluorescent studies were performed to (co)-localize myelin, axonal damage and neuroinflammatory markers. Furthermore, to better characterize the inflammatory process and the status of microglial cells, the mRNA expression of pro- and anti-inflammatory markers was monitored. We also examined whether oxidative stress could be a causative factor in disease onset and progression. Finally, in order to define the role of plasmalogen deficiency in the pathological events, *Gnpat*^−/−^ mice were directly compared with *Nestin-Pex5*^*−/−*^ mice.

## Methods

### Mouse breeding

*Nestin-Pex5* knockout mice and *Gnpat*^*−/−*^ mice were generated as previously described [4,8] and bred into a Swiss Webster background. Tamoxifen inducible mice in which the Cre-ER^TM^ fusion protein is under the control of the ubiquitously active CMV promoter [15] were obtained from The Jackson Laboratory. Tamoxifen was i.p. injected in *CMV-Cre-ER*^*TM*^*-Pex5-loxP* mice at the age of 4 weeks at a dose of 0.2 mg/g body weight. All mice received 5 injections, with one day intervals. Mice were bred in the animal housing facility of the KULeuven, had ad libitum access to water and standard rodent food, and were kept on a 12 h light and dark cycle. All animal experiments were performed in accordance with the “Guidelines for Care and Use of Experimental Animals” and fully approved by the Research Advisory Committee (Research Ethical committee) of the K.U.Leuven (#159/2008).

### Treatment of the mice

*Nestin-Pex5* mice were treated with anti-oxidants as described [11]. N-acetylcysteine (1%) (Acros, Geel, Belgium) was administered via the drinking water (pH adjusted to 3.5) and α-lipoic acid (0.5% w/w) (Sigma-Aldrich, Bornem, Belgium) via the food chow. Treatment was started at the age of 3 weeks for a period of 9 weeks.

### Immunohistochemistry

Paraffin sections were used for immunological detection of almost all antigens, except for C1q, CC-1 and CNP, for which cryosections were used. Stainings were done according to [14]. The sources and concentrations of antibodies are listed in Additional file 1: Table S1. For each age, 3 to 5 individual knockout mice were analyzed and compared with control littermates.

After incubation with primary antibodies overnight, secondary HRP-labeled antibodies were applied during 1 h, followed by fluorescent labeling by the use of the cyanine 2 (FITC) TSA kit (Perkin Elmer Life sciences, Boston, USA). When double or triple immunolabeling was performed, sets of primary and secondary antibodies were sequentially applied. As a second and third fluorescent marker the cyanine 3 and cyanine 5 TSA kits (Perkin-Elmer) were used.

Images were analyzed with a Zeiss CLSM510 confocal laser scanning microscope equipped with a Zeiss axiocam camera.

### Electron microscopy

After perfusion fixation of the corpus callosum with 2.5% glutaraldehyde in 0.1 mol/L phosphate buffer pH 7.2, samples were kept in fixative at 4 ° C overnight. After 1 h post-fixation in 2% osmium tetroxide/0.1 mol/L phosphate buffer pH 7.2 at 4°C, the samples were dehydrated in graded series of alcohol and embedded in epoxy resin. Ultra-thin sections (50 to 60 nm) were cut, stained with uranyl acetate and lead citrate and examined at 50 kV using a Zeiss EM 900 electron microscope (Oberkochen, Germany). Images were recorded digitally with a Jenoptik Progress C14 camera system (Jena, Germany) and operated using Image-Pro express software (Media Cybernetics, USA).

### RNA analysis

qRT-PCR on inflammatory markers was performed as previously described [7]. The assay ID (Applied Biosystems, Halle, Belgium) or the sequence of primers and probes (when custom-made) are listed in Additional file 2: Table S2. FIZZ1 (Mm00445109_m1) and IL10 (Mm00439616_m1) were designed and synthesized by Applied Biosystems and purchased as a premix.

For microarray analysis, the transcriptional profiles of hypothalamus of 10-week-old wild type and *Nestin-Pex5*^*−/−*^ mice (n = 4 per genotype) were analyzed by using the whole genome Affymetrix GeneChip® Mouse Genome 430 2.0 Array as described previously [16]. Labeling of the samples, hybridization, washing and scanning of the chips was carried out at the MicroArray Facility in Leuven (MAF, Leuven, Belgium). The bioinformatics analysis was performed as previously described [16]. The complete dataset is available under GEO record GSE1938.

### *Metabolic analyses*

The TBARS assay to quantify the oxidative stress marker malondialdehyde was performed as decribed [17]. Lipid hydroperoxides were measured with xylenol orange adapted from [18] Dried lipid extracts were redissolved in 0.2 ml chloroform, followed by addition of 0.8 ml reaction mixture (water/methanol 1/3, v/v), containing 32.25 mM H_2_SO_4_, 5 mM FeSO_4_, 250 μM xylenol orange and 31.25 mM butyl hydroxytoluene. After incubation for 30 min, absorbance was read at 560 nm and standardized against cumene hydroperoxide.

## Results

### Demyelination and axon loss in cerebellum of young Nestin-Pex5^−/−^ mice is accompanied with mild gliosis

**Figure 1:**
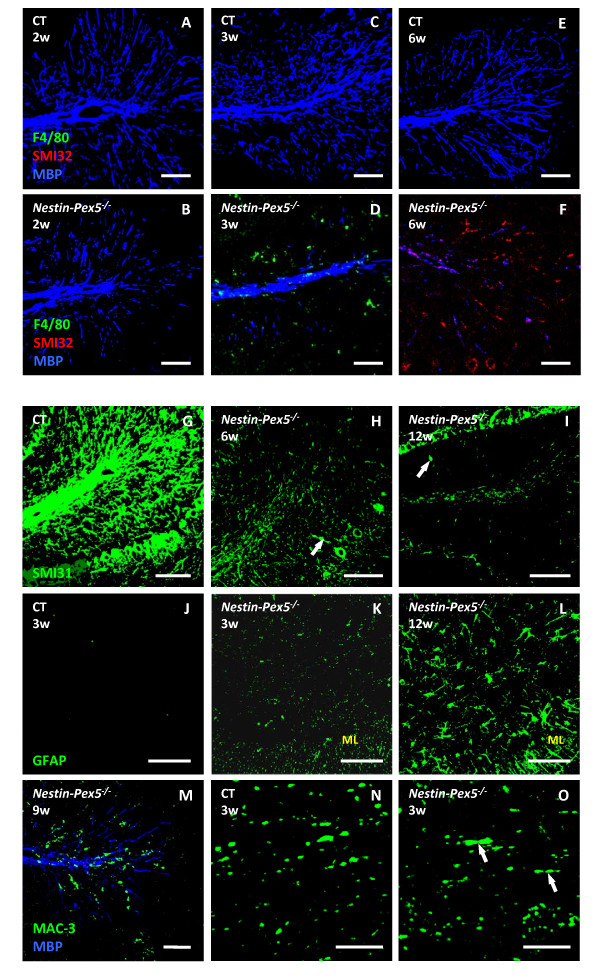
**Early myelin abnormalities but mild microgliosis in cerebellum.** (**A-F**) Immunohistochemistry was performed to visualize microglia (F4/80, green), damaged axons (SMI32, red) and myelin (MBP, blue). Compared to the control mice (A) several fibers in the cerebellar folia of the *Nestin-Pex5*^*−/−*^ already lack myelin at two weeks (B), which was even more pronounced at three weeks (C-D) and six weeks (E-F). SMI32 positive axons, indicative of degeneration, were observed at six weeks (F). (**G-I**) Axonal loss was analyzed by the use of an antibody recognizing healthy phosphorylated axons (SMI31). Decreased SMI31 immunoreactivity was observed at six (H) and 12 weeks (I), in comparison with the control littermates (G). In addition, several axonal swellings were present (H-I, arrows). (**J-L**) Astrocytes were visualized by anti-GFAP, which revealed higher immunoreactivity at three weeks in white matter of the folia, but also in the molecular layer (K). At 12 weeks astrocyte proliferation and activation was even more pronounced (L). (**M**) Double labeling of myelin (MBP, blue) and activated/phagocytotic microglia with MAC-3 (green) in the cerebellum of a nine-week-old *Nestin-Pex5*^*−/−*^ mouse. (**N-O**) An antibody specific for potassium channels (K^+^) was used for the investigation of paranodal structures. Cerebellar axons displayed an abnormal distribution of potassium channels (O, arrows), as compared to the strict juxtaparanodal localization in the control (N). All panels of each row were stained with the same antibodies. Scale bars: A-M: 100 μm; N-O: 10 μm. **GFAP: glial fibrillary acidic protein, K**^**+**^**: potassium, MAC: macrophage, MBP: myelin basic protein, SMI: Sternberger Monoclonals Inc.**

To allow co-localization of microgliosis, myelin abnormalities and axonal damage in *Nestin-Pex5* knockout mice, triple immunofluorescent stainings were performed with anti-F4/80, anti-MBP and either anti-APP (axonal swellings, not shown) or SMI32 (nonphosphorylated neurofilaments, a sign of axonal degeneration). At 3 weeks, when myelin formation is completed, all *Nestin-Pex5*^*−/−*^ mice showed strongly reduced MBP staining of the cerebellar white matter in the folia, indicative of a lack of normal myelin (Figure 1C–D). At the age of 2 weeks, the difference with control mice was less clear although some fibers already lacked myelin in the cerebellar folia (Figure 1A–B). With increasing age (Figure 1E–F), MBP staining of cerebellar axons further diminished, which was in accordance with the appearance of degraded MBP (deMBP) (Additional file 3: Figure S1A–C). Thus, there seems to be both a developmental and a stability problem for cerebellar myelin of *Nestin-Pex5* knockout mice. Damage to cerebellar axons became apparent at 6 weeks by staining unphosphorylated neurofilaments (Figure 1F). In addition, axonal swellings were seen with the neurofilament marker SMI31 (Figure 1H, arrow), which also revealed marked axonal loss from the age of 6 weeks on, in comparison with the wild type littermates (Figure 1G–I). At all stages, only a few F4/80 positive microglial cells were associated with these fibers lacking myelin (Figure 1A–F). Phagocytotic activity of microglia was confirmed by increased staining for the macrophage marker MAC-3 (Figure 1M) and for the lysosomal enzyme cathepsin D (not shown). Besides microglia, also astrocytes take part in the neuroinflammatory cascade. Moderately increased GFAP immunoreactivity was observed at 3 weeks in the white matter of the cerebellar folia and in Bergmann glial cells of the molecular layer (Figure 1J–K). At 12 weeks astrocytes had markedly proliferated and showed a swollen morphology, indicative for astrocyte activation (Figure 1L).

It is well known that paranodes and juxtaparanodes get disorganized along demyelinated axons which according to recent reports might even be an early event in Multiple Sclerosis [19,20]. For this reason, paranodes and juxtaparanodes were visualized by antibodies against caspr and potassium channels respectively. Already at the age of 3 weeks, potassium channels were more spread out over cerebellar fibers, in comparison to the characteristic juxtaparanodal distribution in control mice (Figure 1N–O).

Also in the cerebellar peduncles demyelination (Additional file 4: Figure S2 A–E) and axonal degeneration (Additional file 4: Figure S2C–D, arrows and F–H) takes place, respectively starting at 6 and 9 weeks and aggravating with age. In contrast to the cerebellar folia, marked microgliosis and macrophage activity was observed at 12 weeks (Additional file 4: Figure S2E–F).

### Cortex: Loss of myelin in the absence of a strong microglial response

**Figure 2:**
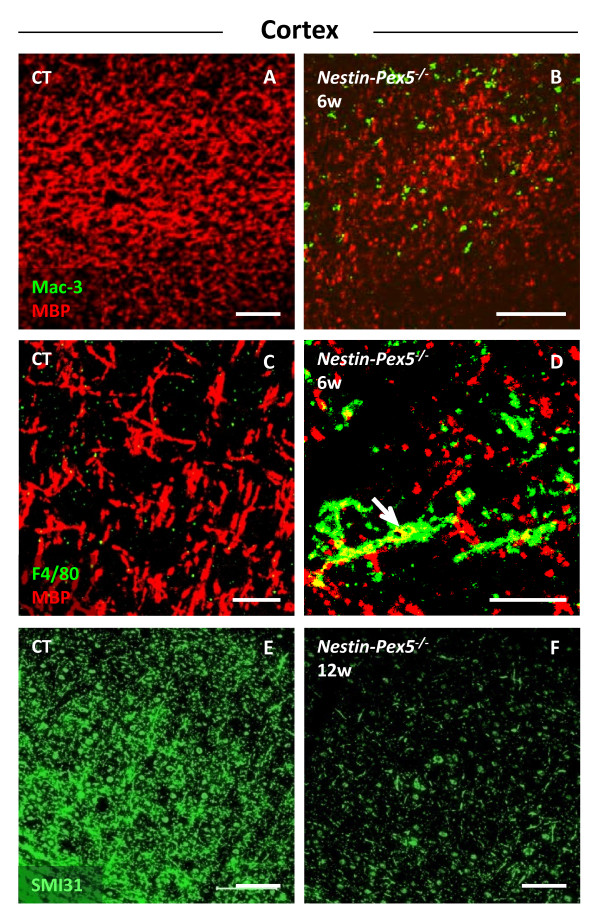
**Myelin loss and mild microgliosis in the cortex.** (**A-D**) Double immunolabeling of myelin (MBP, red) and activated microglia (MAC-3, green) or microglia (F4/80, green), respectively. Cortical demyelination was detected in a subset of *Nestin-Pex5*^*−/−*^ mice at six weeks (B and D), but a strong microglial reaction was never seen (B and D). However, the observed microglial cells were MAC-3 positive, which indicates phagocytotic activity (B) and some microglia seem to contain myelin (D, arrow). (**E-F**) Loss of axons, represented as a decreased SMI31 immunoreactivity, was clearly detectable in the cortex of 12-week-old knockout mice (F), when compared to the control mice (E). All panels of each row were stained with the same antibodies. Scale bars: A-C and G-I: 100 μm; D-F: 25 μm. MBP, myelin basic protein.

The development of pathology in the cortex strongly varied from mouse to mouse but at the age of 12 weeks virtually all mice displayed a massive loss of myelin and axons. A subset of mice displayed demyelination and microgliosis between the age of 3 and 9 weeks, whereas others seemed to be spared at this stage (Figure 2A–D; Additional file 3: Figure S1G–I). Microgliosis is never pronounced, although the few microglial cells are MAC-3 positive (Figure 2B), are swollen and some of these cells contain MBP (Figure 2D, arrow). Likewise astrogliosis is mild at all ages analyzed (data not shown). Using anti-APP or SMI32 staining no axonal swellings were detected in the cortex. However, despite the absence of these signs of axonal damage, extensive axonal loss was observed with SMI31 immunohistochemistry in a subset of mice aged 3 to 9 weeks and from 12 weeks on in all mice (Figure 2E–F).

### Brain stem and spinal cord: Strong correlation between microglia activation and demyelination

**Figure 3:**
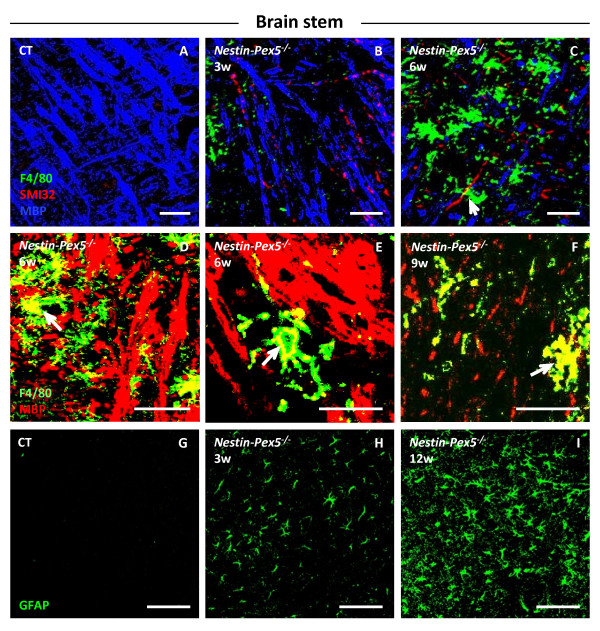
**Association of severe demyelination with extensive microgliosis in the brain stem.** (**A-C**) Triple staining of microglia (F4/80, green), damaged axons (SMI32, red) and myelin (MBP, blue). Already at the age of three and six weeks demyelinated and degenerating axons are observed in a subset of *Nestin-Pex5*^*−/−*^ mice (B-C), in contrast with the control littermates (A). Microglial cells are observed in association with these demyelinated axons (C, arrow). (**D-F**) Higher magnification of the double labeling of myelin (MBP, red) and microglia (F4/80, green) in the brain stem of six- to nine-week-old *Nestin-Pex5*^*−/−*^ mice. The observed microglial cells have a swollen appearance and several of them contain MBP-positive myelin debris (D-F, arrows). (**G-I**) The anti-GFAP antibody was used to analyze astrogliosis, which is mild at three weeks (H), but strongly pronounced at 12 weeks (I), compared with the control mice (G). All panels of each row were stained with the same antibodies. Scale bars: A-D and G-I: 100 μm; E-F: 50 μm. GFAP, glial fibrillary acid protein; MBP, myelin basic protein.

In brain stem, both demyelination and axonal injury were seen at a young age, but the onset varied between 3 and 6 weeks (Figure 3A–C). Early demyelination was confirmed by deMBP staining, which was also seen at higher ages (Additional file 3: Figure S1D–F). Damaged SMI32 positive axons almost always lacked a MBP positive myelin sheet (Figure 3B–C). Moreover, proliferation and activation of microglia appeared from the age of 3 weeks and became more pronounced than in cerebellum. Microglial cells were often seen in the proximity of damaged axons (Figure 3C, arrow) and often contained myelin debris (Figure 3D–F, arrows). Astrocyte proliferation was weakly present from 3 to 9 weeks (not shown) while at 12 weeks both astrocyte proliferation and activation were pronounced (Figure 3G–H). A similar onset and progression of pathology was observed in the cervical part of the spinal cord (not shown).

### Inflammatory demyelination in corpus callosum

**Figure 4:**
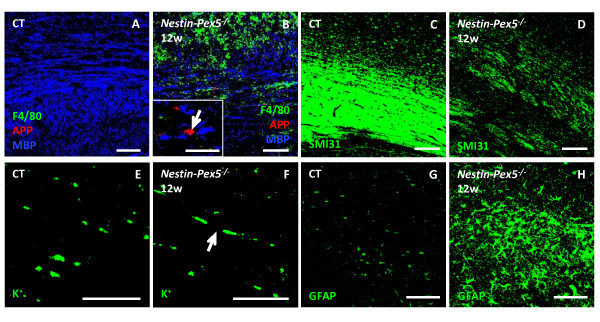
**Corpus callosum is affected later than other brain regions.** (**A-B**) Microglia (F4/80, green), axonal swellings (APP, red) or damage (SMI32, red) and myelin (MBP, blue) were co-labeled on sagittal brain section. At six weeks no pathology was observed yet in the corpus callosum, but at 12 weeks demyelination was pronounced and associated with microgliosis (B) and axonal swellings (Inset in B, arrow). (**C-D**) Healthy axons were massively lost at 12 weeks, as shown by decreased SMI31 immunoreactivity (D), compared to control mice (C). (**E-F**) Potassium channels (K^+^) were immunolabeled to visualize the juxtaparanodes. In the 12-week-old *Nestin-Pex5*^*−/−*^ mice these juxtaparanodes displayed a broader distribution (F, arrow), in comparison with the control mice (E). (**G-H**) Astrocytes were labeled with anti-GFAP. Astrogliosis is strongly pronounced at 12 weeks (H), in comparison with the control mouse (G). Scale bars: A-D: 100 μm; G-H: 10 μm; Inset in B: 12 μm. APP: amyloid precursor protein, GFAP: glial fibrillary acidic protein, K^+^: potassium, MBP: myelin basic protein, SMI: Sternberger monoclonals Inc.

Although signs of demyelination and axon damage could already be detected in corpus callosum of some 3- to 9-week-old mice, a major loss of myelin and axons was noticed in all 12-week-old mice (Figure 4A–D; Additional file 3: Figure S1J–L). Similar as in the brain stem, axonal swellings were mostly detected where myelin is absent (Inset in Figure 4B, arrow). At this age myelin loss was accompanied with disorganized juxtaparanodes based on potassium channel stainings (Figure 4E–F, arrow). Important to note is that demyelination in the corpus callosum was always accompanied by extensive microgliosis (Figure 4B) and phagocytotic activity (not shown). A low grade of astrogliosis is seen from 3 to 9 weeks (not shown), but this worsened significantly from 12 weeks on, and became more pronounced than in all other brain areas (Figure 4H).

**Figure 5:**
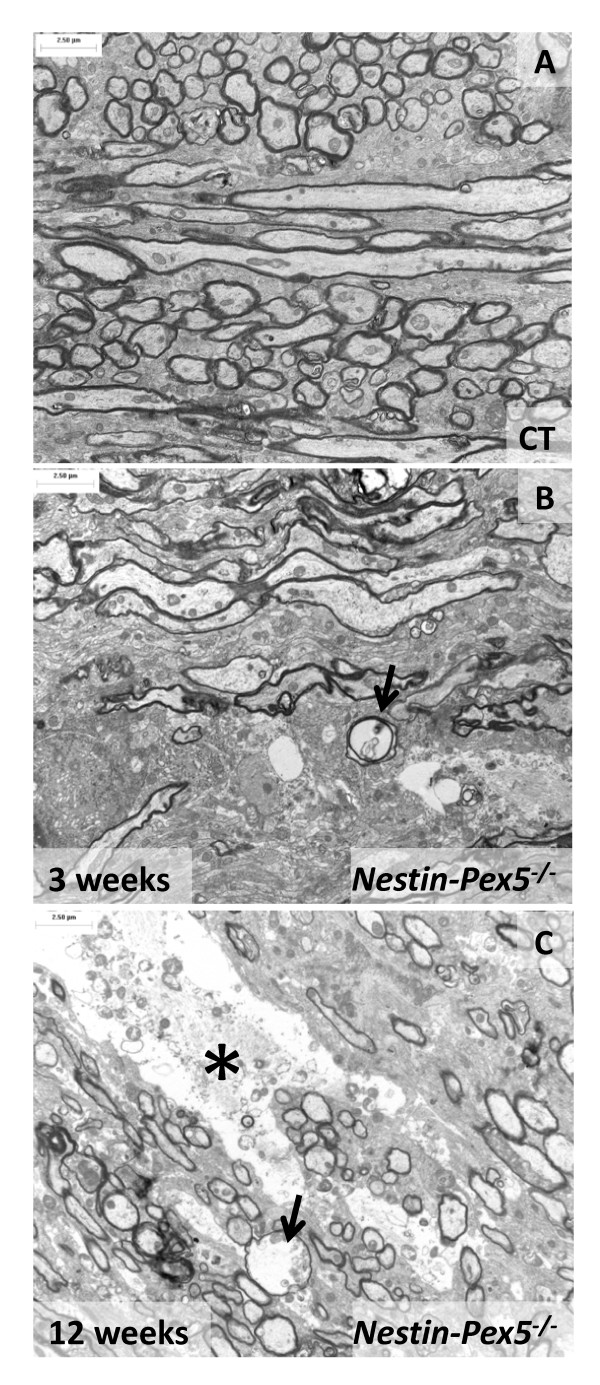
**Ultrastructure of myelin and axons in corpus callosum.** (**A**) Cross and longitudinal sections of nerve fibers of control mice display a regular and compact myelin sheath. (**B**) In the corpus callosum of three-week-old *Nestin-Pex5*^*−/−*^ mice many axons are still myelinated while a subset already lacks a myelin sheath. The arrow in (B) indicates a swollen necrotic axon, surrounded by a normally appearing myelin sheath. (**C**) In 12-week-old *Nestin-Pex5* knockout mice demyelination is even more pronounced than at three weeks. Similarly as at three weeks, an edematous necrotic axon, which is enwrapped by an apparently normal myelin sheath (arrow), is detected. In addition, large necrotic areas are observed (asterisk).

Electron microscopy (EM) of the corpus callosum of *Nestin-Pex5* knockout mice was performed to complement the fluorescence microscopy. The ultrastructure of the corpus callosum of control mice (Figure 5A) showed cross and longitudinal sections of axons with a regular and compact myelin sheath. *Nestin-Pex5* knockout mice of 3 weeks (Figure 5B) showed axons with normal myelin sheaths but also denuded axons. Some axons displayed a swollen necrotic cytoplasm which was still enwrapped by a clear myelin sheath (arrow). At 12 weeks (Figure 5C) axonal injury became more apparent showing completely necrotic areas with remnants of cytoplasmic organelles and cytoskeleton elements (asterisk). Again it is important to note that several oedematous axons are still myelinated (arrow). Taken together, degeneration of myelinated axons in corpus callosum can already be detected by EM analysis at the age of 3 weeks. This strongly aggravates with age which then can be visualized with immunofluorescent stainings.

### Activation of the immune system in nestin-Pex5 knockout mice

To obtain an overall picture of altered gene expression in peroxisome deficient brain, microarray analysis was performed. Hypothalamus tissue of 10-week-old mice was chosen because this brain area can be easily delineated and pathology is progressing at this age based on histological observations. The KEGG pathways that were most significantly up regulated are all related to immune responses, including cytokine-cytokine receptor interaction, transendothelial migration, antigen processing and presentation, and toll-like receptor signaling pathways (Table 1). In order to confirm these data, the expression of a selection of inflammatory markers was evaluated in other brain areas and at other time points by qRT-PCR and by immunofluorescence.

**Table 1:** Microarray analysis on hypothalamus of 10-week-old *Nestin-Pex5*^*−/−*^ mice

**Cytokine-cytokine receptor interaction**		
**Complement**		**Fold change**
*C3ar1*	complement component 3a receptor 1	11.28^***^
*C1qb*	complement component 1, q subcomponent, beta polypeptide	9.4^***^
*C4b*	complement component 4B	8.47^***^
*C1qa*	complement component 1, q subcomponent, alpha polypeptide	7.64^***^
**Chemokines**		
*Cxcl13*	chemokine (C-X-C motif) ligand 13	16.8^***^
*Ccl6*	chemokine (C-C motif) ligand 6	11.93^***^
*Ccl3*	chemokine (C-C motif) ligand 3	8.13^***^
*IL12b*	Interleukin 12b	5.67^***^
**Leukocyte transedothelial migration**		
*Itgb2*	Integrin beta 2	5.57^***^
*Itgam*	Integrin alpha M	3.56^***^
*ICAM1*	Intercellular adhesion molecule 1	4.12^***^
*VCAM1*	Vascular cell adhesion molecule 1	1.52^***^
**Antigen processing and presentation**		
*H2-K1*	Histocompatibility 2, K1, K region	5.57^***^
*H2-D1*	Histocompatibility 2, D region locus 1	3.56^***^
*Fcgr2b*	Fc receptor, IgG, low affinity IIb	6.21^***^
*Fcgr3*	Fc receptor, IgG, low affinity III	4.41^***^
*P2Y6*	Pyrimidinergic receptor P2Y, G-protein coupled 6	5.22^***^
*B2m*	beta-2 microglobulin	3.06^***^
*Ctse*	cathepsin E	1.52^***^
**Toll-like receptor signaling pathway**		
*TLR2*	Toll-like receptor 2	4.70^***^
*TLR1*	Toll-like receptor 1	4.43^***^
*TLR7*	Toll-like receptor 7	1.98^***^
*TLR4*	Toll-like receptor 4	1.70^***^
**Anti-inflammatory markers**		
*IL4*	Interleukin 4	**0.93**
*IL10*	Interleukin 10	**1.03**
*IL13*	Interleukin 13	**1.01**
*Arg1*	Arginase-1	**1.13**
*TGFβ*	Transforming growth factor β	2.54^***^
*HO-1*	Heme oxygenase-1	2.37^***^
**Anti-oxidantia**		
*Cat*	Catalase	1.23^*^
*MnSOD*	Manganese superoxide dismutase	**0.95**
*Prdx6*	Peroxiredoxin 6	1.34^***^
*Prdx1*	Peroxiredoxin 1	1.12^*^
*Gpx1*	Glutathione peroxidase 1	1.25^***^

Only the most affected KEGG pathways are represented. Values are given as means of 4 independent samples. * p < 0.05, *** p < 0.001.

**Figure 6:**
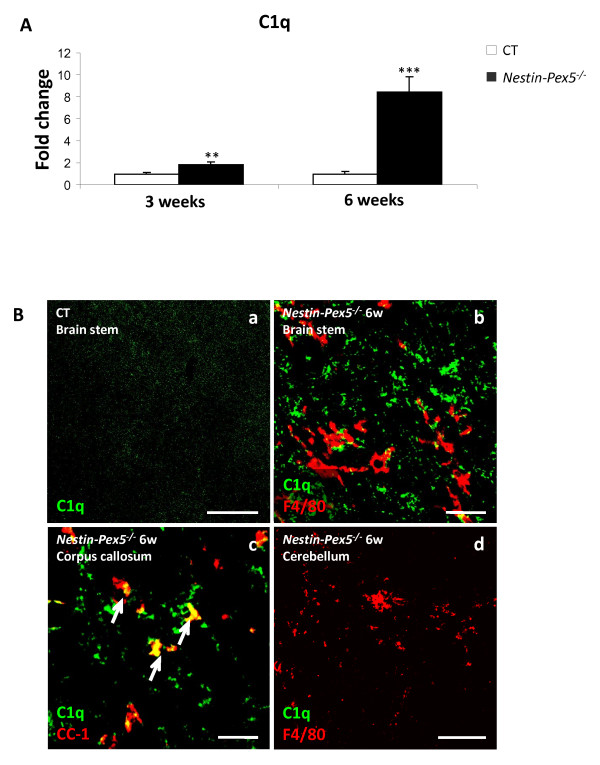
***C1q*expression is an early response in Nestin-Pex5**^**−/− **^**brain.** (**A**) C1q expression levels were quantified by qRT-PCR. Up regulation of mRNA levels was already significant in the corpus callosum of the three-week-old *Nestin-Pex5*^*−/−*^, although this was much more pronounced at six weeks. Expression levels were normalized to β-actin expression. Values are given as means ± SEM of four independent samples. ** *P* < 0.01, *** *P* < 0.001 (Student’s *t*-test). (**B-E**) C1q was also investigated by immunohistochemistry. At six weeks, C1q immunoreactivity was observed in the corpus callosum (D) and brain stem (C), but not in the cerebellum (E). Double labeling of C1q (green) and microglia (F4/80, red) or oligodendrocytes (CC-1, red), respectively, was performed to determine the cellular localization of the complement protein. C1q was not present in microglia (B and D), but in a subset of oligodendrocytes (C). Scale bars: B and E: 50 μm; C and D: 20 μm. SEM, standard error of the mean.

Based on the microarray results, mRNA levels of several components of the complement system are highly up regulated. C1q, one of the most markedly up regulated genes, is the first subcomponent of the classical complement system and was further investigated by qRT-PCR. Strikingly, C1q mRNA levels were already significantly elevated at 3 weeks, and increased dramatically with age in corpus callosum (Figure 6A). In contrast, in cerebellum C1q transcripts were only mildly up regulated (Table 2). In order to localize C1q expression, immunohistochemistry on sagittal brain sections was performed. C1q immunoreactivity was already increased at the age of 3 and 6 weeks in brain stem (Figure 6C) and at 6 weeks in corpus callosum (Figure 6D), preceding the detection of microgliosis by F4/80 in these brain areas. In accordance with the mRNA data, no C1q deposits were found in cerebellum (Figure 6E). As it was previously shown that C1q can be expressed by different cell types, co-localization studies were performed. There is no overlap of C1q with F4/80, suggesting that this complement factor is not expressed by microglia at 6 weeks (Figure 6C and E). Partial overlap is seen with CC-1 (Figure 6D, arrows), a marker for oligodendrocyte cell bodies. The cellular C1q deposits did not overlap with myelin sheaths or axons, but based on the shape of the immunoreactive structures the majority of C1q was present on the surface of neuronal cell bodies (not shown), although we could not determine the localization by double stainings.

**Table 2:** Fold change of mRNA levels of inflammatory mediators incorpus callosum and cerebellum of 6-week-old *Nestin-Pex5* mice

**Nestin-Pex5 6 weeks**		
**Fold change**	**Corpus callosum**	**Cerebellum**
**TNFα**	28.96 ± 6.27^***^	17.15 ± 8.85
**C1q**	8.40 ± 1.43^***^	2.94 ± 1.45
**TLR2**	11.91 ± 3.26^***^	12.42 ± 6.10
**Mpeg1**	13.64 ± 6.38^***^	10.63 ± 0.34^***^
**TGFβ**	2.83 ± 0.18^***^	3.38 ± 0.5^**^
**IL10**	30.92 ± 10.65^*^	3.18 ± 3.10
**Arg1**	1.78 ± 0.08^**^	2.78 ± 1.11
**Fizz1**	13.31 ± 8.59	1.62 ± 0.50

Values represent means of 4 independent samples. * p < 0.05, ** p < 0.01, *** p < 0.001. Expression levels were normalized to β-actin expression.

**Figure 7:**
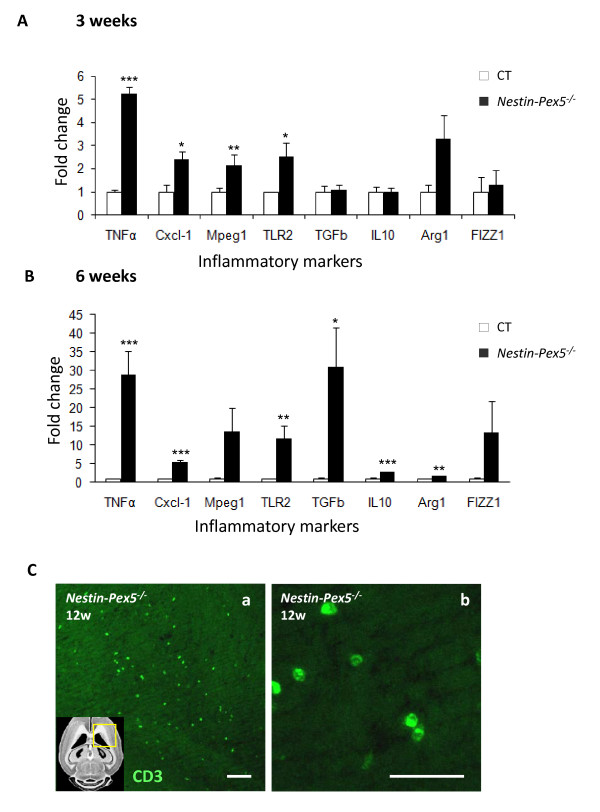
**Up regulation of pro- and anti-inflammatory markers in Nestin-Pex5**^**−/− **^**mice.** (**A-B**) In the corpus callosum of three- and six-week-old *Nestin-Pex5*^*−/−*^ mice mRNA levels of several pro- and anti-inflammatory markers were quantified by qRT-PCR. (A) TNFα***, Cxcl-1*, Mpeg1** and TLR2* were all significantly up regulated in the corpus callosum of three-week-old knockout mice. The anti-inflammatory markers TGFβ, IL10, arginase 1 and FIZZ1 were not up regulated at three weeks. (B) At six weeks the pro-inflammatory markers showed much higher expression levels, compared to three-week-old *Nestin-Pex5* knockout mice. Also expression levels of the anti-inflammatory markers TGFβ, IL10 and arginase 1 were significantly increased at this age. Values represent means ± SEM of four independent samples, * *P* < 0.05, ** *P* < 0.01, *** *P* < 0.001 (Student’s *t*-test). Expression levels were normalized to β-actin expression. (**C-D**) By immunohistochemistry infiltration of CD3 positive lymphocytes was observed in 12-week-old *Nestin-Pex5*^*−/−*^mice, mainly in the corpus callosum. Scale bars: C: 50 μm; D: 25 μm. CD: cluster of differentiation, Cxcl: chemokine (C-X-C motif) ligand, FIZZ: found in inflammatory zone, IL: interleukin, Mpeg: macrophage expressed gene, SEM: standard error of the mean, TGF: transforming growth factor, TLR: toll like receptor.

Other pro-inflammatory markers, including TNFα, the toll-like receptor TLR2 and the chemokine Cxcl-1, were analyzed by qRT-PCR in corpus callosum of 3- and 6-week-old mice (Figure 7A-B) and in cerebellum of 6-week-old mice (Table 2). All these inflammatory mediators were highly up regulated even before demyelination, axonal pathology and microgliosis are detectable by fluorescence microscopy. This is particularly obvious in corpus callosum, where expression of pro-inflammatory markers is strongly increased at 6 weeks, whereas the peak of microglia activation only occurs from 12 weeks on.

Importantly, the microarray data also pointed to phagocytotic activity of inflammatory cells in the brain of 10-week-old *Nestin-Pex5*^−/−^ mice (Table 1), confirming histological results. Receptors involved in the binding and ingestion of pathogens, including Fc receptors, complement receptors and P_2_Y_6_ were highly up regulated (Table 1). The latter receptor recognizes the nucleotide UDP released from injured neurons and stimulates phagocytosis [21]. Furthermore, expression of Mpeg1, a macrophage marker [22] and of lysosomal proteins, were significantly increased. Examples are cathepsin D (2.54 fold up regulated), Lamp2 (1.57 fold) and hexosaminidase B (3.17 fold). This was further confirmed by qRT-PCR on Mpeg1 in other brain regions and at earlier time points (Figure 7A and B; Table 2).

In a normal inflammatory process, a pro-inflammatory phase is followed by a resolution phase, during which microglial cells produce anti-inflammatory markers [23,24]. According to microarray analysis only TGFβ and Heme oxygenase-1 were up regulated (2.54 and 2.37 fold respectively) (Table 2). However, additional anti-inflammatory markers [25] were analysed by qRT-PCR analysis in corpus callosum of 3- and 6-week-old mice. As Arginase 1, Fizz1, IL10 and TGFβ were all significantly up regulated in 6-week-old *Nestin-Pex5*^*−/−*^ mice, it appears that the pro-inflammatory response is accompanied by an anti-inflammatory reaction. In cerebellum of 6-week-old *Nestin-Pex5*^*−/−*^ mice, only TGFβ is significantly elevated, the other anti-inflammatory markers were not (Table 2).

The microarray results further revealed the up regulation of genes involved in transendothelial migration of blood-derived inflammatory cells into the brain. To verify increased infiltration, immunohistochemistry with an antibody recognizing T-cells (CD3) was performed. At the age of 12 weeks a limited number of T-cells were noticed in corpus callosum of *Nestin-Pex5*^−/−^ mice (Figure 7C–D).

Together, these data indicate that the innate immune system is activated at a very early stage in peroxisome deficient brain, generating a strong and persistent pro-inflammatory response which could not be stopped by anti-inflammatory mechanisms. Secondarily, the adaptive immune system is activated.

### Oxidative stress

**Figure 8:**
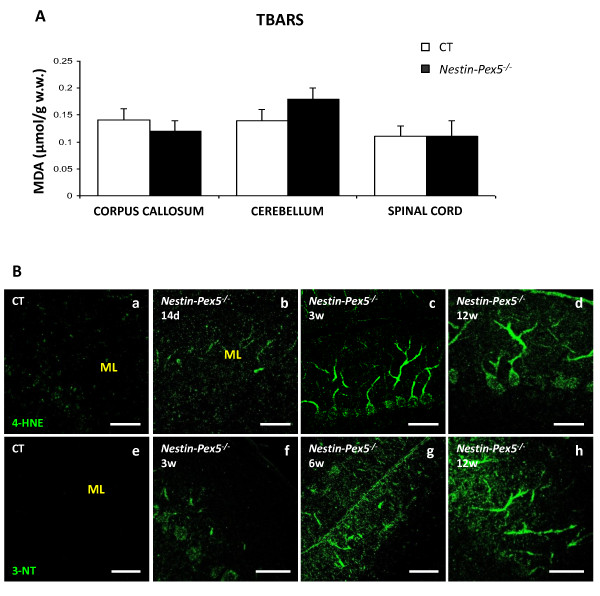
**Oxidative stress markers are only present in Purkinje cells.** (**A**) End products of lipid peroxidation, predominantly malondialdehyde, were quantified by the TBARS assay. No differences between 9- to 12-week-old *Nestin-Pex5*^*−/−*^ and control littermates were observed in the corpus callosum, cerebellum or spinal cord. Values are given as means ± SEM of four independent samples of each genotype (Student’s *t*-test). (**B-I**) The markers 4-HNE (B-E) and 3-nitrotyrosine (3-NT) (F-I) were evaluated by immunohistochemistry. 4-HNE was slightly observable in Purkinje cells of two-week-old *Nestin-Pex5*^*−/−*^ mice (C), compared with the control littermates (B). The amount of 4-HNE present in Purkinje cells was higher at three (D) and 12 weeks (E). Low 3-NT immunoreactivity was observed at three weeks in Purkinje cells (G), but increased with age (H-I). Scale bars: B-F and G: 100 μm; G and I: 50 μm. 4-HNE, 4-hydroxynonenal; SEM, standard error of the mean; TBARS, thiobarbituric acid reactive substances.

Because it was reported that oxidative stress precedes the axon pathology in spinal cord of *Abcd1* knockout mice, lacking the peroxisomal transporter ABCD1 [10,11], several experiments were conducted to investigate whether oxidative stress occurs in *Nestin-Pex5* knockouts. The TBARS assay was performed to detect end markers of lipid peroxidation, predominantly malondialdehyde, in the CNS of *Nestin-Pex5* mice (Figure 8A). Furthermore, lipid hydroperoxides were quantified using xylenol orange. No evidence for lipid peroxidation was found in corpus callosum, cerebellum and spinal cord of 9- to 12-week-old knockout mice (data not shown).

As it was reported that immunohistochemistry is a more sensitive procedure to detect markers of oxidative stress in brain [26], stainings were performed with antibodies reacting with 4-hydroxynonenal (4-HNE). Slightly higher 4-HNE immunoreactivity was detected in Purkinje cells already at postnatal day 14 (Figure 8B–C) which increased with age (Figure 8D–E). In addition, 4-HNE staining was sporadically observed along cortical fibers and in the corpus callosum but not in all mice and not at all ages tested (not shown). In all the other brain regions and cell types, there was no difference in immunoreactivity between *Nestin-Pex5*^*−/−*^ and control mice. To confirm these findings, oxidative damage to proteins was examined by staining with antibodies to 3-nitrotyrosine [27]. Immunoreactivity was also elevated in Purkinje cells of *Nestin-Pex5*^*−/−*^ mice, starting at 3 weeks (Figure 8F–G) and increasing with age (Figure 8H–I), but not in other brain regions. Interestingly, according to microarray data there is no up regulation of the cytokine iNOS in *Nestin-Pex5*^*−/−*^ mice, which was confirmed by qRT-PCR analysis in corpus callosum of 6-week-old mice (not shown). In agreement with iNOS, also mRNA levels of NADPH oxidase were not altered in the microarray. However, at the age of 5 months significantly higher levels of iNOS were detected in corpus callosum (2.35 fold, p < 0.05). At this age, also 4-HNE immunoreactivity was observed in corpus callosum of a subset of *Nestin-Pex5*^*−/−*^ mice.

Up regulation of anti-oxidative enzymes is another indicator of increased oxidative stress. According to microarray analysis the peroxiredoxins 1 and 6, catalase and glutathione peroxidase 1 were mildly but significantly up regulated, whereas several other ones such as MnSOD were not. MnSOD immunoreactivity was also not different between brains of *Nestin-Pex5*^*−/−*^ mice and control littermates (Table 1). Strongly increased catalase immunoreactivity in *Nestin-Pex5* knockouts was already reported [4].

To rule out that a low level of oxidative stress, that was not detectable with the used methods, contributes to the pathology, *Nestin-Pex5*^*−/−*^ mice were treated from the age of 3 weeks for a period of 9 weeks with an anti-oxidant mixture of α-lipoic acid (0.5% w/w in food chow) and N-acetylcysteine (1% in drinking water). This treatment did not lead to improvement of the motor performance and health status of the mice. Microscopically, catalase levels were not reduced and 4-HNE was still detected in Purkinje cells of treated *Nestin-Pex5*^*−/−*^ mice. Thus, anti-oxidative treatment did not affect the phenotype of *Nestin-Pex5* knockout mice.

### No neuroinflammation in the gnpat knockout model

One of the major peroxisomal metabolic pathways is the synthesis of ether lipids, including plasmalogens which are very enriched in brain myelin. It was previously reported that cerebellar and neocortical dysmyelination occurs in juvenile *Gnpat* knockout mice, a model with selective ether lipid deficiency [9]. In order to examine the contribution of deficient ether lipid synthesis to the pathology of *Nestin-Pex5* knockout mice, the two models were directly compared. These two models show a similar premature death rate, with a critical period between P1 (postnatal day 1) and weaning when 30 – 50% of the knockouts die. However, *Gnpat* knockout mice can survive more than 20 months, whereas *Nestin-Pex5*^−/−^ mice always die before the age of 6 months. First we analyzed the levels of plasmalogens in the brains of both models at 3 weeks. Whereas plasmalogen levels are 15-fold lower in the *Nestin-Pex5* knockout brain in comparison to controls (0.6 nmol/mg tissue vs 9.8 nmol/mg) plasmalogen levels were even further reduced below the detection level (< 0.03 nmol/mg tissue) in *Gnpat*^−/−^ mice.

**Figure 9:**
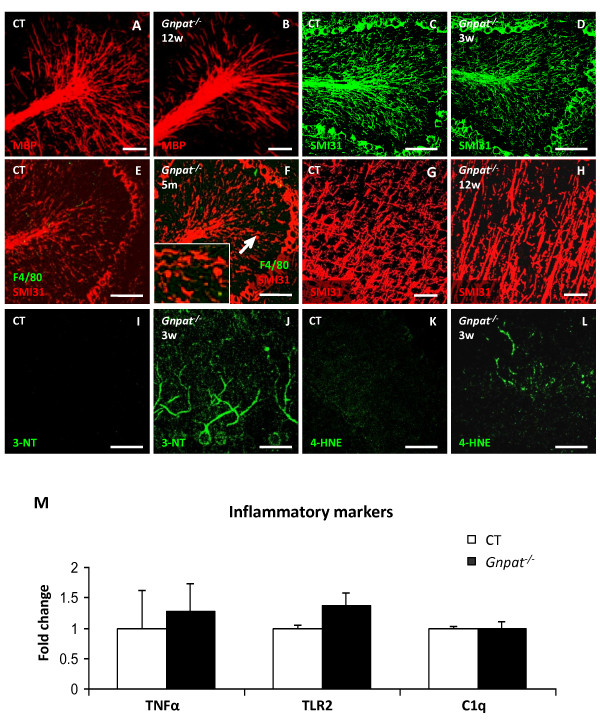
**Cerebellar and cortical dysmyelination in*Gnpat***^***−/− ***^**mice in the absence of microglia activation.** (**A-L**) Immunohistochemistry was performed to investigate myelin (MBP), axonal loss (SMI31), microgliosis (F4/80) and oxidative stress (4-HNE and 3-nitrotyrosine). *Gnpat*^−/−^ mice displayed a decreased amount of myelin in the cerebellar folia at three months (B), compared with the control littermates (A). Cortical demyelination was observed in a subset of three-month-old *Gnpat* knockout mice (G-H). Axonal loss was very mild at three weeks (C-D) and five months (E-F) and also a few axonal swellings were detectable with SMI31 (red) at five months (F, arrow and inset). Microgliosis (F4/80, green) was absent (F). 3-Nitrotyrosine (3-NT) and 4-HNE positive Purkinje cells were seen in three-week-old *Gnpat*^−/−^ mice (J, L), in comparison with the control mice (I, **K**). Scale bars: A-F and I-L: 100 μm; G-H: 50 μm. (**M**) Quantification of mRNA levels of the pro-inflammatory markers TNFα, TLR2 and C1q by qRT-PCR. Expression levels were not elevated in four- to five-month-old *Gnpat* knockout mice. Values represent means ± SEM of four independent samples. Expression levels were normalized to β-actin expression. 4-HNE, 4-hydroxynonenal; TLR2, toll-like receptor 2; SEM, standard error of the mean.

Next, immunhistochemical analyses were performed on brain sections of *Gnpat*^−/−^ mice at the age of 3 and 12 weeks and 5 months. At the age of 3 weeks, MBP staining is diminished in cerebellum, similar to the reduced staining in 3-week-old *Nestin-Pex5* knockouts. However, at 12 weeks and 5 months, myelin levels did not further decrease in *Gnpat*^−/−^ mice (Figure 9A–B). Subtle axonal loss was observed in this brain area at 3 weeks (Figure 9C) by SMI31 staining and occasionally at 12 weeks and 5 months (Figure 9E–F). As previously reported [9], axonal swellings were observed on Purkinje cell axons (Figure 9F, arrow and inset), the abundance of which strongly correlated with the ataxic behavior of *Gnpat*^−/−^ mice.

In the cortex, some *Gnpat* knockout mice (aged 3 weeks – 5 months) also displayed reduced MBP immunoreactivity (Figure 9G–H), which however never reached the level of cortical demyelination of 12-week-old *Nestin-Pex5*^*−/−*^ mice. In general, the lack of myelin and axonal loss in the *Gnpat*^−/−^ is clearly less drastic, compared to the *Nestin-Pex5*^*−/−*^ mice and restricted to the neocortex and cerebellum at all ages analyzed. Microgliosis was not observed in the knockout mice whereas a slightly increased number of astrocytes was noticed at 3 months in the white matter of the cerebellar folia and at 3 and 5 months in the brain stem (not shown). To investigate whether the innate immune system was activated, inflammatory markers were analyzed in the forebrain and brain stem of 4-month-old *Gnpat*^−/−^ mice, but, remarkably, pro-inflammatory cytokines were not induced (Figure 9M).

Markers of oxidative stress were investigated by immunohistochemistry. Similar to the *Nestin-Pex5*^*−/−*^ mice, an increased immunoreactivity for 3-nitrotyrosine (Figure 9I–J) and a slightly higher reactivity for 4-HNE was detected in Purkinje cells of 3-week-old *Gnpat*^*−/−*^ mice (Figure 10  K-L). At 12 weeks and 5 months, only a subset of knockout brains stained positive for 3-nitrotyrosine and 4-HNE in Purkinje cells. Higher protein levels of catalase were also detected in the *Gnpat* knockout, but this was only observed in the white matter and Bergmann glial cells of the cerebellum and not in the cerebrum. This is in contrast to *Nestin-Pex5*^*−/−*^ where higher catalase levels were seen throughout the brain [4]. Summarizing, despite a more severe deficiency of plasmalogens as compared to *Nestin-Pex5*^*−/−*^ mice, *Gnpat*^−/−^ mice do not develop an inflammatory phenotype, nor extensive demyelination and axonal loss.

### Inactivation of peroxisomes after development causes similar pathology as the nestin-Pex5^−/−^

To exclude that the brain phenotype of *Nestin-Pex5*^*−/−*^ mice is somehow a consequence of mild developmental delays [5], a mouse model was investigated in which CNS peroxisomes were inactivated at a later time point. To this end, CMV-Cre-ER^TM^ mice, in which the expression of Cre-recombinase fused to a mutated estrogen receptor ligand binding domain is driven by the ubiquitously active CMV promoter, were bred with *Pex5-loxP* mice. After administration of tamoxifen at the age of 4 weeks, the Cre recombinase fusion protein becomes activated causing *Pex5* recombination. As previously shown (paper submitted, supplemental Figure 3), wild type PEX5p was not completely lost in all tissues, but inactivation was particularly effective in brain (less than 20% wild type Pex5 mRNA). Based on the cytosolic pattern of catalase, predominantly astrocytes and oligodendrocytes and some microglia were devoid of functional peroxisomes, whereas most neurons still contained a peroxisomal catalase staining (Additional file 5: Figure S3, arrows).

These mice developed significant motor problems from the age of 5 months which gradually aggravated with age, leading to death from the age of 8 months. This is similar to *Nestin-Pex5*^*−/−*^ mice although with a later onset and a slower progression (Additional file 6: Figure S4A). Immunohistochemistry at 3, 5 and 8 months of age revealed that brain pathologies developed in the same sequence and in the same brain regions as in *Nestin-Pex5*^*−/−*^ mice but with later onset. Microgliosis was mildly present in a subset of knockouts at 3 months, mainly in the cerebellum and the brain stem (not shown). However, it became more pronounced at 5 and 8 months in those brain regions (cerebellum, Additional file 4: Figure S4Ba-c). Microgliosis coincided with demyelination (Additional file 4: Figure S4Ba-c) and axonal damage (Additional file 4: Figure S4 Bb), axonal loss (Additional file 4: Figure S4 Bd–e) and swellings (Additional file 4: Figure S4Bf, arrow), which is the most pronounced at 8 months of age in these areas. Demyelination was confirmed by the presence of deMBP in the cerebellar folia (not shown). As in the *Nestin-Pex5*^*−/−*^ mice the corpus callosum was affected later than other brain regions, displaying microgliosis between 5 and 8 months, but no demyelination or axonal damage (Additional file 4: Figure S4Bg–i).

Thus, loss of functional peroxisomes from brain at any age induces a neurodegenerative program accompanied with a strong inflammatory response.

## Discussion

Absence of functional peroxisomes in the postnatal mouse CNS causes a neurodegenerative phenotype that ultimately leads to extensive axon loss and early death. This closely mimics pathologies in mild peroxisome biogenesis disorders and the cerebral form of X-ALD. We here investigated the involvement of micro- and astroglia activation, metabolic factors and oxidative stress in the onset and progression of myelin and axon loss triggered by peroxisome inactivity.

In all demyelinating areas, increased numbers of microglia were present. Remarkably, in cerebellum and cortex microglia were never very abundant, whereas they were strongly represented in brain stem starting in the juvenile period and they massively invaded the demyelinating corpus callosum of 12-week-old mice. Also of note is that astroglial activation lagged behind microglia proliferation, as described before in other neurodegenerative diseases [28]. Many microglia displayed features of phagocytosing cells including a swollen appearance, sometimes containing MBP-positive myelin debris and surrounding a demyelinating fiber. This was also confirmed by increased transcripts for macrophage markers such as Mpeg1 and lysosomal enzymes.

According to recent insights, the number and shape of microglia does not predict the type of immunological reaction that is developing. Besides the classical pro-inflammatory microglia, macrophages occur that are involved in the resolution of inflammation which have been named “alternatively activated” or “deactivated” [29]. In all brain regions, markers of a pro-inflammatory response, such as TNFα, IL6 and IL1β were highly up regulated [30]. Although both markers of alternative microglial activation (Arginase 1, Fizz1) and of acquired deactivation (IL10, TGFβ) were up regulated in peroxisome deficient corpus callosum before the peak of microglial activation occurred, this could not halt the progression of inflammation.

It was remarkable that the induction of markers of the innate immune system occurred at a very early stage in the disease process, within 3 weeks after birth. Strikingly, it was recently reported that peroxisome deficiency in *Pex1* mutant Drosophila larvae also caused up regulation of genes involved in innate immunity, further supporting that peroxisome deficiency modulates immunological responses [31]. In addition, peroxisomes were shown to be involved in the generation of defense factors following a viral infection and thus participate in antiviral signaling [32]. The precise role of peroxisomes in shaping the innate immune responses will need to be further elucidated.

With regard to neuronal morphology, we did not find much evidence for cell death [4] but there was progressive axonal loss throughout the CNS which was already very obvious at the age of 12 weeks based on neurofilament staining with antibody SMI31. At this and earlier ages, axonal swellings (detected by staining with anti-APP) and damage (detected by staining unphosphorylated neurofilaments with SMI32) could be visualized, but these stainings strongly underestimated ongoing axonal degeneration. By EM analysis, some axonal irregularities were already observed at the age of 3 weeks, illustrating the very early onset of the degenerative phenotype. The progressive axonal loss coincided with severe and progressive motor and cognitive decline, which we reported before [4]. In this respect, it is interesting to mention that axonal injury is currently considered as the most important cause of clinical disabilities in MS [33,34].

With the exception of a variable onset of lesions in the cortex, the development of brain pathology was consistent from mouse to mouse. It was also recapitulated, but delayed in time, in mice in which peroxisomes were deleted from the CNS in the juvenile period after completion of myelination. This ruled out the possibility that the observed anomalies were a late consequence of peroxisome ablation during development. Our present findings that demyelination in cerebellum precedes white matter abnormalities in cerebrum is in striking agreement with reports in mildly affected peroxisome biogenesis patients [35,36] in which regressive changes predominate over developmental anomalies. By MRI analysis the first foci of white matter abnormalities were found in the central cerebellar area, additional lesions were often seen in the brain stem whereas the cerebral hemispheres were affected later. This distribution of white matter lesions is clearly different from the typical pattern in X-ALD in which the splenium of the corpus callosum is affected first. Remarkably, the leukoencephalopathy in PBD patients was not predictive for the clinical outcome [35] suggesting that additional pathology causes the psychomotor retardation. In addition, patients have been identified with milder *PEX* mutations, displaying normal mental capacities, but developing progressive ataxia, caused by cerebellar atrophy [36-38]. Why cerebellar neurons are selectively affected in these longer surviving patients is not clear.

A direct comparison of the lesions in *Nestin-Pex5*^*−/−*^ mice with those in *Gnpat*^−/−^ mice allowed to determine the contribution of hampered ether phospholipid synthesis to the observed pathologies. Ether phospholipids are quintessential products of peroxisomal metabolism that are very enriched in the CNS and myelin under the form of plasmalogens. *Gnpat*^−/−^ mice were previously shown to have myelin deficits in cerebellum and swellings on Purkinje cell axons [9]. Although the pattern of hypomyelination, axonal swellings and axonal loss was very similar in cerebellum of juvenile *Gnpat* and *Nestin-Pex5* knockout mice, with increasing age, *Gnpat*^−/−^ mice did not further lose myelin in cerebellum, nor in other brain areas. Also in rhizomelic chondrodysplasia punctata RCDP patients, abnormalities in white matter were reported which were however rather located in supratentorial areas [39]. In sharp contrast with *Nestin-Pex5* knockout mice no phagocytotic microglia nor reactive astrocytes were detected at the age of 6 months throughout the *Gnpat*^−/−^ brain which was further confirmed by the absence of pro-inflammatory markers. These findings are in accordance with the pathology observed in the *Pex7*^*−/−*^ mouse model for RCDP type 1. At the age of 9 – 11 months the latter mice do not display a reactive response of microglia [40] but they show mild astrocytosis. Likewise, in RCDP patients non-inflammatory dysmyelination and occasionally astrocytosis, but no inflammatory demyelination has been reported [41]. A mild degree of gliosis is observed in a case of *GNPAT* deficiency [42]. In view of the inflammatory demyelination in patients with peroxisomal β-oxidation deficiency [43], the latter metabolic defect is likely facilitating the inflammatory response in brain lacking peroxisomes. Extensive gliosis was indeed observed in *Mfp2* knockout mice although the pro- versus anti-inflammatory character was not investigated yet. A role for plasmalogen shortage as a factor synergizing with peroxisomal β-oxidation defects to induce inflammation can however not be excluded. Indeed, microglia activation was observed in the *Pex7:Abcd1* double knockout mouse model but not in the single knockouts [40].

An important question is whether the axon injury culminating in axonal loss is due to myelin abnormalities, to neuroinflammation, to other factors, or a combination of these. We can indeed exclude that this is the consequence of peroxisome deficiency within neurons, as we previously showed that neuron selective inactivation of *Pex5* did not cause a neurodegenerative phenotype [7]. Based on fluorescence microscopy, and particularly obvious in cerebellum, axonal swellings and degeneration mostly appear after demyelination, indicating that the lack of myelin evokes loss of the denuded axon. On the other hand, as previously reported and now confirmed at younger ages, ultrastructural analysis showed degenerating axons that were still surrounded by a full myelin sheath, indicating that alternative mechanisms may also be operating. The milder phenotype of *Gnpat* knockout mice, in which an inflammatory reaction is absent, strongly indicates that the early and severe activation of the innate immune system contributes to the neurodegenerative phenotype in *Nestin-Pex5*^*−/−*^ mice. The detrimental impact of neuroinflammation on axonal survival is well established. In MS patients, axonal loss is closely related to the degree of inflammation in the active lesions [34,44]. In addition, it should be kept in mind that peroxisomes play an essential role in oligodendrocytes, because the selective loss of functional peroxisomes from these cells also results in inflammatory demyelination [6]. In this respect the early expression of the complement component C1q on oligodendrocytes and neurons might be indicative of an endangered response which secondarily evokes inflammation. Indeed, although the complement system can be stimulated by both the innate and the adaptive immune system, it here seems to play a role in the early activation of innate immunity as the adaptive system is only involved much later with infiltrating T cells. Also in a mouse model for spinal cord injury, C1q is expressed on axons and oligodendrocytes [45]. In Alzheimer disease, C1q causes activation of C3d, and via the complement pathway it can activate microglia with a damaging effect on myelin and axons [46]. Although the relationship between peroxisomes and innate immunity needs further investigation, we speculate that metabolic abnormalities, likely related to peroxisomal β-oxidation defects, initiate an early activation of the innate immune system, which together with abnormalities in the formation and maintenance of myelin creates an environment which is detrimental for axons.

The absence of oxidative stress in young *Nestin-Pex5*^*−/−*^ mice is in contrast with recent findings in *Abcd1* knockout mice, a model for the AMN form of X-ALD. In these mice oxidative damage to proteins was detected in spinal cord but not in brain, preceding by several months axonal damage in spinal cord. The crucial role of this oxidative stress for the pathogenesis was further proven as anti-oxidative therapy could prevent the degenerative phenotype. Increased concentrations of the very long chain fatty acid, C_26:0_ is thought to be the metabolic factor initiating oxidative stress [47]. It remains unclear why this occurs in spinal cord and not in brain with *Abcd1* deficiency and likewise, why C_26:0_ accumulation in *Nestin-Pex5* knockout mice [4] does not trigger an oxidative stress response. In fact, in patients with *ABCD1* deficiency, lipid peroxidation in plasma was higher in the AMN phenotype than in cerebral ALD and in asymptomatic patients [48]. On the other hand, in PBD patients oxidative stress markers were not increased in plasma or urine [49]. Taken together, the causal relationship between oxidative stress and inflammatory demyelination is currently unclear and we have no evidence that degeneration in the peroxisome deficient brain is triggered or boosted by the development of oxidative stress.

## Conclusions

A lack of plasmalogens contributes to abnormalities in the formation and stability of myelin but not to the uncontrolled pro-inflammatory response in the brain of *Nestin-Pex5*^*−/−*^ mice. This neurotoxic environment leads to axonal damage and a chronic progression of neurodegeneration. It remains to be investigated whether the neurodegenerative phenotype can be halted by blocking the inflammatory response.

## **Abbreviations**

3-NT, 3-nitrotyrosine; 4-HNE, 4-hydroxynonenal; Abcd1, ATP-binding cassette, subfamily D, member 1; AMN, adrenomyeloneuropathy; APP, amyloid precursor protein; CNP, cyclic nucleotide phosphodiesterase; CNS, central nervous system; CMV, cytomegalovirus; Ccl, chemokine (C-C motif) ligand; Cxcl, chemokine (C-X-C motif) ligand; deMBP, degraded MBP; EM, electron microscopy; ER, estrogen receptor; FITC, fluorescein isothiocyanate; FIZZ, Found in inflammatory zone; GFAP, glial fibrillary acidic protein; Gnpat, glyceronephosphate O-acyltransferase; HRP, horseradish peroxidase; IgG, immunoglobulin G; IL, interleukin; K+, potassium; iNOS, inducible nitric oxide synthase; KEGG, Kyoto Encyclopedia of Genes and Genomes; Lamp, lysosome associated membrane protein; LRM, lipid raft microdomain; MAC-3, macrophage; MBP, myelin basic protein; Mfp-2, multifunctional protein 2; MnSOD, manganese superoxide dismutase; Mpeg, macrophage expressed gene; MS, multiple sclerosis; NADPH, nicotinamide adenine dinucleotide phosphate; PBD, peroxisome biogenesis disorder; Pex, peroxin; RCDP, rhizomelic chondrodysplasia punctata; SEM, standard error of the mean; SMI, Sternberger monoclonals inc; TBARS, thiobarbituric acid reactive substances; TGF, transforming growth factor; TLR, Toll like receptor; TNF, tumor necrosis factor; TSA, tyramide signal amplification; UDP, uridine diphosphate; X-ALD, X-linked adrenoleukodystrophy.

## Competing interests

The author(s) declare that they have no competing interests.

## Authors’ contributions

AB and SV carried out the phenotyping of the mice, RD carried out the EM analysis, PPVV supervised the biochemical work, WJ generated the *Gnpat* knockout mice, AB and MB drafted the manuscript. All authors read and approved the final manuscript.

## Supplementary Material

Additional file 1 **Table S1.** Antibodies used for immunohistochemistry. Click here for file

Additional file 2 **Table S2.** List of primers and probes used for real-time PCR. Click here for file

Additional file 3 **Figure S1.** Demyelination in different brain areas visualized by deMBP. To visualize disintegration of myelin, immunohistochemistry was performed with an antibody recognizing degraded MBP (deMBP). DeMBP was present from 3 weeks on in the cerebellum of *Nestin-Pex5*^*-/-*^ mice (A–C) and also in the brain stem (D–F). In addition, deMBP was seen in the cortex, although only at 3 weeks (G) and virtually not at higher ages (H–I). The corpus callosum displays slight deMBP immunoreactivity at 6 weeks (J) and 9 weeks (K), but much more pronounced at 12 weeks (L). Scale bars: 100 μm. Click here for file

Additional file 4 **Figure S2.** Pathology in the cerebellar peduncles. (A–C) Triple immunolabeling of microglia (F4/80, green), degrading axons (SMI32, red) and myelin (MBP, blue) was performed on brain sections of 6- to 9-week-old *Nestin-Pex5*^*-/-*^ mice. At 6 and 9 weeks only sporadically a microglial cell (B, arrow) and a SMI32 positive axon was observed (C, arrows). MBP loss was not clearly seen at these ages with MBP. (D) 9-week-old *Nestin-Pex5*^*-/-*^ mice displayed deMBP immunoreactivity (green) in the cerebellar peduncles, indicating demyelination. In addition, damaged axons were observed in the same region with SMI32 (red, arrows). (E–F) The cerebellar peduncles of 12-week-old *Nestin-Pex5* mice were also examined by triple staining for microglia (F4/80, green) or activated microglial (MAC-3, green), axonal damage (SMI32, red) and myelin (MBP, blue). At 12 weeks microgliosis (E) and microglia activation (F) but also demyelination and axonal degeneration was more pronounced than at earlier ages. (G–H) SMI31 immunoreactivity was decreased in the knockout (H) compared with the control mice (G), which represents axonal loss. Scale bars: 100 μm. Click here for file

Additional file 5 **Figure S3.** Analysis of peroxisome inactivation in tamoxifen induced CMV-Tx-Pex5^-/-^ mice. Inactivation of PEX5p was investigated by the visualization of catalase on brain sections of *CMV-Tx-Pex5* mice. Under normal circumstances catalase colocalizes with peroxisomes, resulting in a punctuate staining pattern. PEX5p deficient cells are not able to import catalase, resulting in ectopic localization of catalase in the cytosol and consequently a green uniform staining of the cell. Astrocytes (GFAP, red) and oligodendrocytes (CC-1, red) display the cytosolic staining pattern of catalase (A and B, arrows) whereas neurons (MAP-2, red) still contain the punctuate pattern (C, arrows). In microglial cells (F4/80, red, arrows) neither a cytosolic nor a punctuate pattern can be recognized, probably due to low levels of catalase (D). Scale bars: A–B: 50 μm; C–D: 20 μm. Click here for file

Additional file 6 **Figure S4.** Brain pathology in mice with deletion of functional peroxisomes in adulthood. (A) Motor performance of CMV-Tx-Pex5^-/-^ mice was tested by the use of an accelerating rotarod. At 3 months motor performance of knockout mice was indistinguishable from the control littermates, but decreased significantly between 5 and 8 months. At least 4 animals were investigated per age and per genotype. ** p < 0.01, *** p < 0.001 (student’s *t*-test). (B) (a–c) Labeling of microglia (F4/80, green), degenerating axons (SMI32, red) and myelin (MBP, blue) on brain sections of *CMV-Tx-Pex5* mice. The cerebellum displayed mild demyelination at 5 and 8 months (a–c) and several SMI32 positive axons at 5 months (b). (d–f) Axonal loss was detectable as a decreased SMI31 immunoreactivity in 8-month-old *CMV-Tx-Pex5*^*-/-*^ compared to control mice. (f) Axonal swellings were also observed with SMI31 (f, arrow). (g–i) F4/80 (green), SMI32 (red) and MBP (blue) triple staining of corpus callosum. Microgliosis was detected in corpus callosum of 5- and 8-month-old knockout mice, but there was no evidence for demyelination nor axonal degeneration (g-i). Click here for file
