# Supplementary material for: Peroxisome deficiency but not the defect in ether lipid synthesis causes activation of the innate immune system and axonal loss in the central nervous system
Source: J Neuroinflammation. 2012 Mar 29;9:61. doi: 10.1186/1742-2094-9-61 (PMC3419640; doi:10.1186/1742-2094-9-61)
Supplement: Additional file 1 — Table S1. Antibodies used for immunohistochemistry. [file 1742-2094-9-61-S1.docx]

Supplementary table 1: Antibodies used for immunohistochemistry.

| **Identity** | **Antibody** | **Antigen retrieval*** | **Dilution** | **Company** |
| --- | --- | --- | --- | --- |
| 3-nitrotyrosine | 3-NT | / | 1/100 | Millipore, Temecula, USA |
| 4-hydroxynonenal | 4-HNE | / | 1/500 | Merck, Darmstadt, Germany |
| Axonal swellings | APP | Trypsinization | 1/200 | Chemicon, Temecula, USA |
| Complement C1q | C1q | / | 1/500 | Abcam, Cambridge, UK |
| Paranodes | Caspr | / | 1/200 | Abcam, Cambridge, UK |
| Anti-oxidant | Catalase | Tryspinization | 1/100 | Rockland, Gilbertsville, USA |
| Oligodendrocytes | CC-1 | Microwave | 1/50 | Calbiochem, San Diego, USA |
| Myelin | CNP | / | 1/100 | Sternberger, Baltimore, USA |
| Degraded myelin | deMBP | Microwave | 1/1000 | Biognost, Heule, Belgium |
| Microglia | F4/80 | / | 1/500 | Serotec, Oxford, UK |
| Astrocytes | GFAP | Trypsinization | 1/200 | Sigma-Aldrich, Bornem, Belgium |
| Juxtaparanodes | K^+^ | Microwave | 1/100 | Alomone lab, Jerusalem, Israel |
| Activated microglia | MAC-3 | / | 1/100 | BD biosciences, Franklin Lakes, USA |
| Neuronal dendrites | MAP-2 | Tryspinization | 1/100 | Chemicon, Temecula, USA |
| Myelin | MBP | Microwave | 1/500 | Millipore, Temecula, USA |
| Phosphorylated neurofilament | SMI31 | Microwave | 1/500 | Sternberger, Baltimore, USA |
| Unphosphorylated neurofilament | SMI32 | Microwave | 1/2000 | Sternberger, Baltimore, USA |

* Trypsinization: incubation of slides for 30 seconds with 0.05% trypsin. Microwave: 1 minute maximum, 9 minutes middle power in citrate buffer (pH 6).
