# Supplementary material for: Peroxisome deficiency but not the defect in ether lipid synthesis causes activation of the innate immune system and axonal loss in the central nervous system
Source: J Neuroinflammation. 2012 Mar 29;9:61. doi: 10.1186/1742-2094-9-61 (PMC3419640; doi:10.1186/1742-2094-9-61)
Supplement: Additional file 2 — Table S2. List of primers and probes used for real-time PCR. [file 1742-2094-9-61-S2.docx]

Supplementary Table 2: List of primers and probes used for real-time PCR

| **Gene** | **Oligonucleotides** |
| --- | --- |
| β-actin | For: AGAGGGAAATCGTGCGTGAC  Rev: CAATAGTGATGACCTGGCCGT  Probe: CACTGCCGCATCCTCTTCCTCCC |
| C1q | For : AAGGACTGAAGGGCGTGAAA  Rev : CACGTTGCCAAGCGTCATT  Probe : CAATATCAGGGACCAGCCCCGGC |
| TNFα | For : ATCCGCGACGTGGAACTG  Rev : ACCGCCTGGAGTTCTGGAA  Probe : CAGAAGAGGCACTCCCCCAAAAGATG |
| TLR2 | For : GCCCTTCTCCTGTTGATCTTGCT  Rev : CGCCCACATCATTCTCAGGTA  Probe : GTGCCCTGTGCCACCATTTCC |
| Cxcl-1 | For: TGCACCCAAACCGAAGTCA  Rev : GAGCTTCAGGGTCAAGGCAAG  Probe : AGCCACACTCAAGAATGGTCGCGA |
| Mpeg1 | For : ATGCCAAGTGTCCTACTGTGTCAA  Rev : CCTGGCTCATAAGAGGTGGTTT  Probe : CTGCTCCCTGTCAGGCTCCCACC |
| iNOS | For : TGGAGAGATTTGCATGACACTCTT  Rev : CCAAGCAAGACTTGGACTTGCA  Probe : CACCACAAGGCCACATCGGATTTCACT |
| Arginase 1 | For : AAAGGAAAGTTCCCAGATGTACCA  Rev : TACGTCTCGCAAGCCAATGTA  Probe : TGACTCCCTGCATATCTGCCAAAGACATC |
