# Supplementary material for: Peroxisome deficiency but not the defect in ether lipid synthesis causes activation of the innate immune system and axonal loss in the central nervous system
Source: J Neuroinflammation. 2012 Mar 29;9:61. doi: 10.1186/1742-2094-9-61 (PMC3419640; doi:10.1186/1742-2094-9-61)
Supplement: Additional file 3 — Figure S1. Demyelination in different brain areas visualized by deMBP. To visualize disintegration of myelin, immunohistochemistry was performed with an antibody recognizing degraded MBP (deMBP). DeMBP was present from 3 weeks on in the cerebellum of Nestin-Pex5-/- mice (A–C) and also in the brain stem (D–F). In addition, deMBP was seen in the cortex, although only at 3 weeks (G) and virtually not at higher ages (H–I). The corpus callosum displays slight deMBP immunoreactivity at 6 weeks (J) and 9 weeks (K), but much more pronounced at 12 weeks (L). Scale bars: 100 μm. [file 1742-2094-9-61-S3.ppt]

## Slide 1
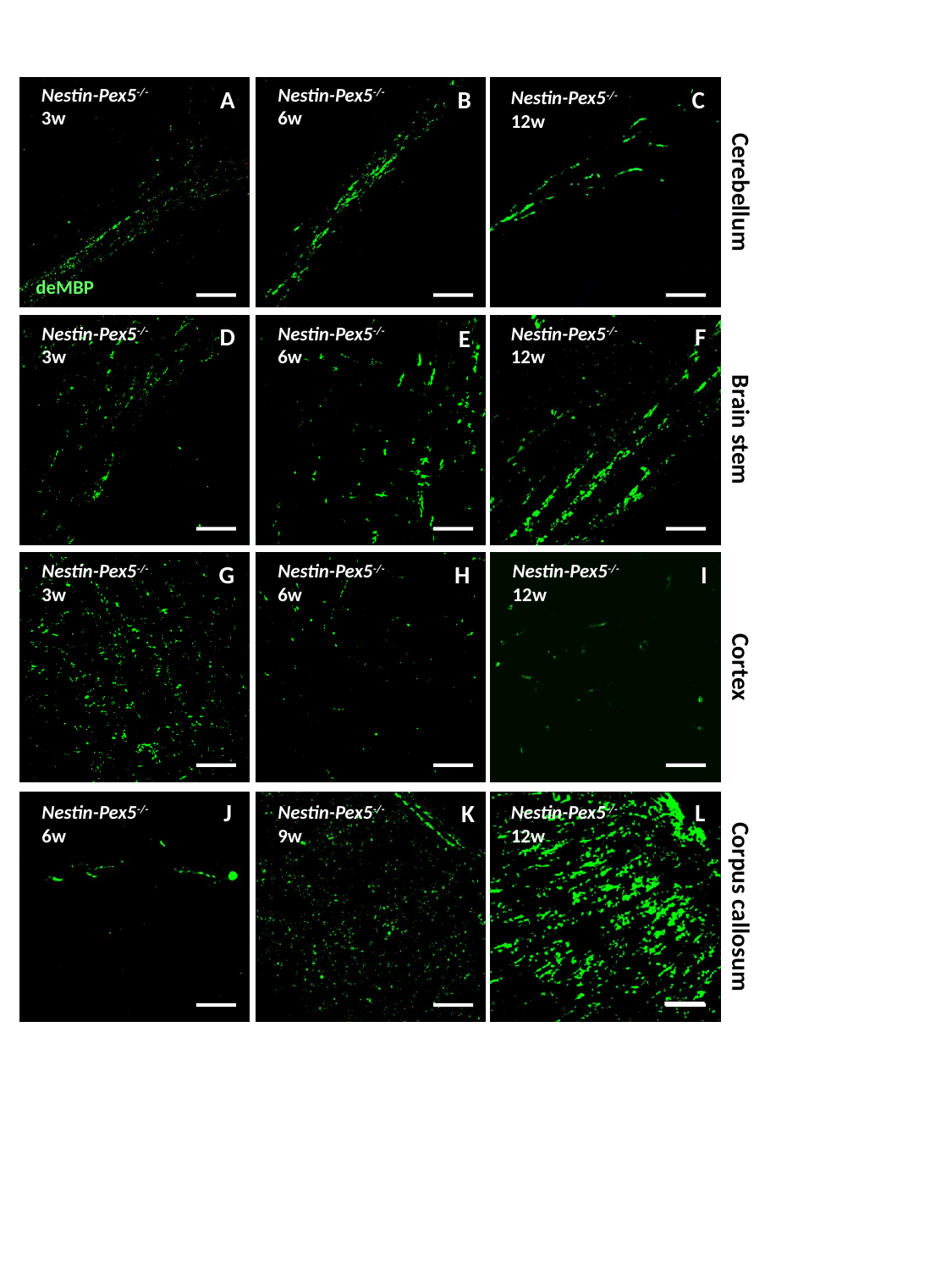

Nestin-Pex5-/-
3w
Nestin-Pex5-/-
6w
A
C
B
Nestin-Pex5-/-
12w
Cerebellum
deMBP
F
Nestin-Pex5-/-
3w
D
Nestin-Pex5-/-
6w
Nestin-Pex5-/-
12w
E
Brain stem
Nestin-Pex5-/-
3w
Nestin-Pex5-/-
6w
Nestin-Pex5-/-
12w
H
G
I
Cortex
J
L
K
Nestin-Pex5-/-
6w
Nestin-Pex5-/-
9w
Nestin-Pex5-/-
12w
Corpus callosum
