# Supplementary material for: Peroxisome deficiency but not the defect in ether lipid synthesis causes activation of the innate immune system and axonal loss in the central nervous system
Source: J Neuroinflammation. 2012 Mar 29;9:61. doi: 10.1186/1742-2094-9-61 (PMC3419640; doi:10.1186/1742-2094-9-61)
Supplement: Additional file 4 — Figure S2. Pathology in the cerebellar peduncles. (A–C) Triple immunolabeling of microglia (F4/80, green), degrading axons (SMI32, red) and myelin (MBP, blue) was performed on brain sections of 6- to 9-week-old Nestin-Pex5-/- mice. At 6 and 9 weeks only sporadically a microglial cell (B, arrow) and a SMI32 positive axon was observed (C, arrows). MBP loss was not clearly seen at these ages with MBP. (D) 9-week-old Nestin-Pex5-/- mice displayed deMBP immunoreactivity (green) in the cerebellar peduncles, indicating demyelination. In addition, damaged axons were observed in the same region with SMI32 (red, arrows). (E–F) The cerebellar peduncles of 12-week-old Nestin-Pex5 mice were also examined by triple staining for microglia (F4/80, green) or activated microglial (MAC-3, green), axonal damage (SMI32, red) and myelin (MBP, blue). At 12 weeks microgliosis (E) and microglia activation (F) but also demyelination and axonal degeneration was more pronounced than at earlier ages. (G–H) SMI31 immunoreactivity was decreased in the knockout (H) compared with the control mice (G), which represents axonal loss. Scale bars: 100 μm. [file 1742-2094-9-61-S4.ppt]

## Slide 1
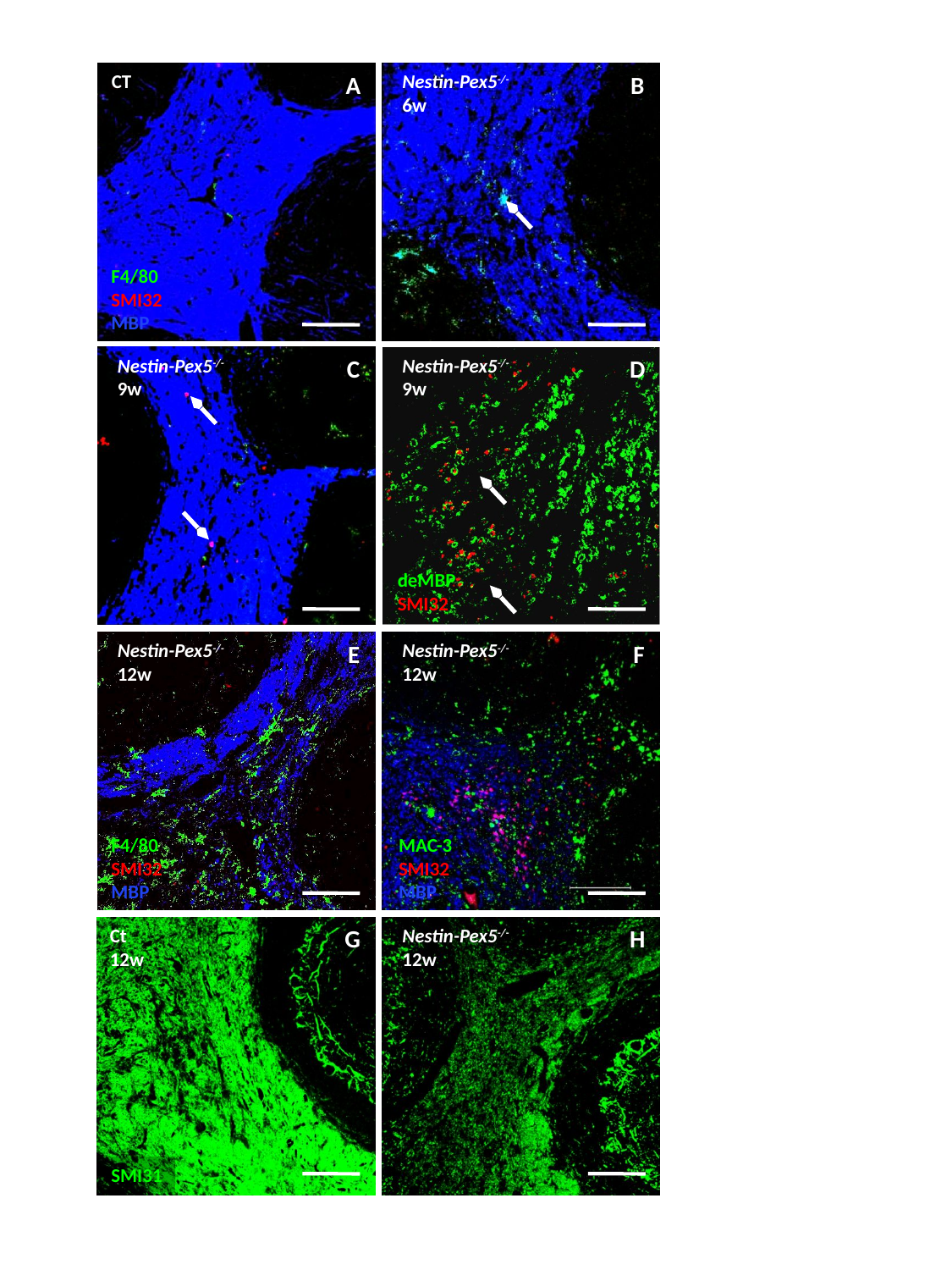

CT
A
Nestin-Pex5-/-
6w
B
F4/80
SMI32
MBP
20 µm
Nestin-Pex5-/-
9w
C
Nestin-Pex5-/-
9w
D
deMBP
SMI32
Nestin-Pex5-/-
12w
E
Nestin-Pex5-/-
12w
F
F4/80
SMI32
MBP
MAC-3
SMI32
MBP
Ct
12w
G
Nestin-Pex5-/-
12w
H
SMI31
