# Supplementary material for: Peroxisome deficiency but not the defect in ether lipid synthesis causes activation of the innate immune system and axonal loss in the central nervous system
Source: J Neuroinflammation. 2012 Mar 29;9:61. doi: 10.1186/1742-2094-9-61 (PMC3419640; doi:10.1186/1742-2094-9-61)
Supplement: Additional file 5 — Figure S3. Analysis of peroxisome inactivation in tamoxifen induced CMV-Tx-Pex5-/- mice. Inactivation of PEX5p was investigated by the visualization of catalase on brain sections of CMV-Tx-Pex5 mice. Under normal circumstances catalase colocalizes with peroxisomes, resulting in a punctuate staining pattern. PEX5p deficient cells are not able to import catalase, resulting in ectopic localization of catalase in the cytosol and consequently a green uniform staining of the cell. Astrocytes (GFAP, red) and oligodendrocytes (CC-1, red) display the cytosolic staining pattern of catalase (A and B, arrows) whereas neurons (MAP-2, red) still contain the punctuate pattern (C, arrows). In microglial cells (F4/80, red, arrows) neither a cytosolic nor a punctuate pattern can be recognized, probably due to low levels of catalase (D). Scale bars: A–B: 50 μm; C–D: 20 μm. [file 1742-2094-9-61-S5.ppt]

## Slide 1
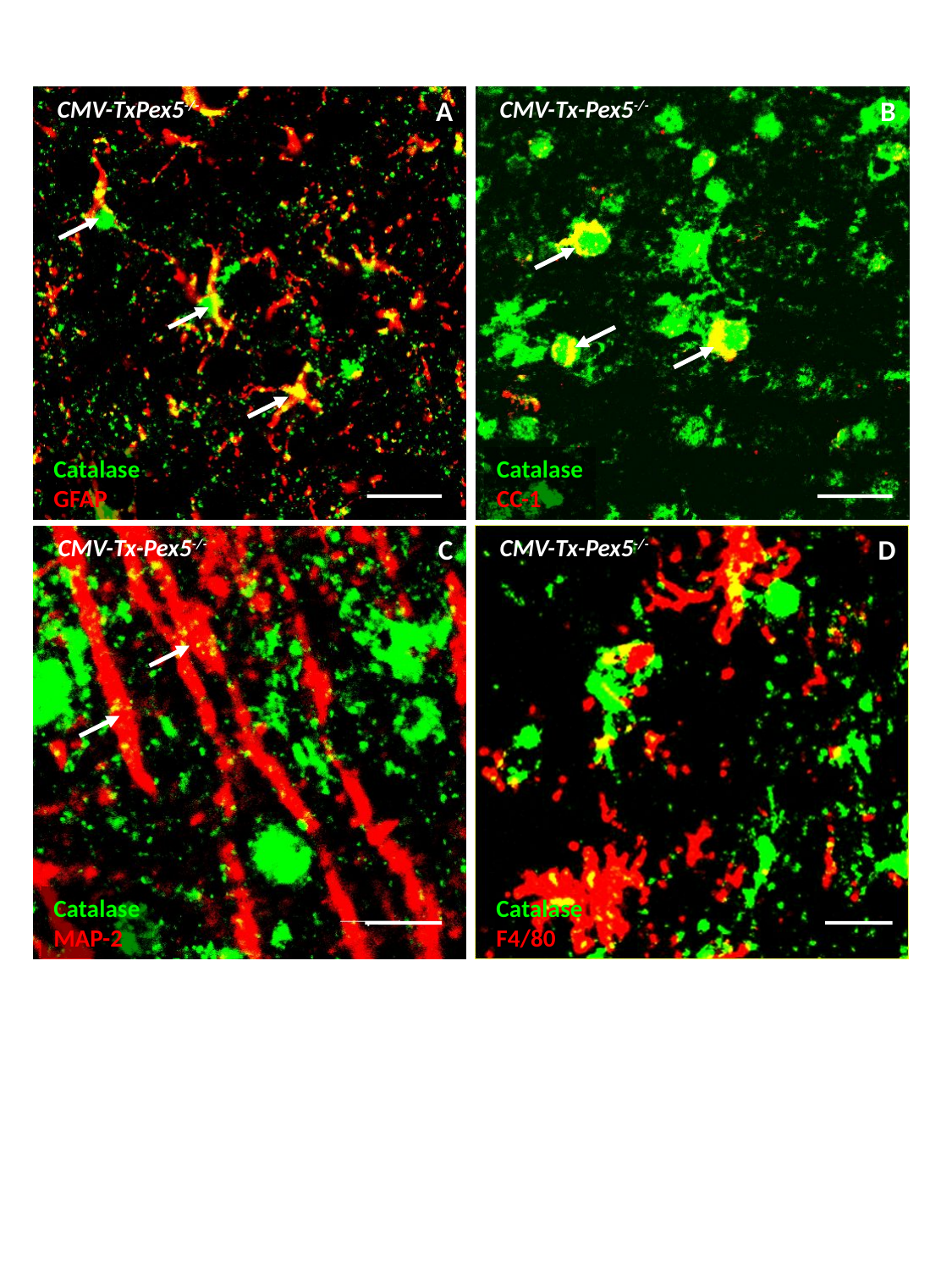

A
CMV-TxPex5-/-
A
CMV-Tx-Pex5-/-
B
Catalase
GFAP
Catalase
CC-1
CMV-Tx-Pex5-/-
C
CMV-Tx-Pex5-/-
D
C
D
Catalase
MAP-2
Catalase
F4/80
