# Supplementary material for: Peroxisome deficiency but not the defect in ether lipid synthesis causes activation of the innate immune system and axonal loss in the central nervous system
Source: J Neuroinflammation. 2012 Mar 29;9:61. doi: 10.1186/1742-2094-9-61 (PMC3419640; doi:10.1186/1742-2094-9-61)
Supplement: Additional file 6 — Figure S4. Brain pathology in mice with deletion of functional peroxisomes in adulthood. (A) Motor performance of CMV-Tx-Pex5-/- mice was tested by the use of an accelerating rotarod. At 3 months motor performance of knockout mice was indistinguishable from the control littermates, but decreased significantly between 5 and 8 months. At least 4 animals were investigated per age and per genotype. ** p < 0.01, *** p < 0.001 (student’s t-test). (B) (a–c) Labeling of microglia (F4/80, green), degenerating axons (SMI32, red) and myelin (MBP, blue) on brain sections of CMV-Tx-Pex5 mice. The cerebellum displayed mild demyelination at 5 and 8 months (a–c) and several SMI32 positive axons at 5 months (b). (d–f) Axonal loss was detectable as a decreased SMI31 immunoreactivity in 8-month-old CMV-Tx-Pex5-/- compared to control mice. (f) Axonal swellings were also observed with SMI31 (f, arrow). (g–i) F4/80 (green), SMI32 (red) and MBP (blue) triple staining of corpus callosum. Microgliosis was detected in corpus callosum of 5- and 8-month-old knockout mice, but there was no evidence for demyelination nor axonal degeneration (g-i). [file 1742-2094-9-61-S6.ppt]

## Slide 1
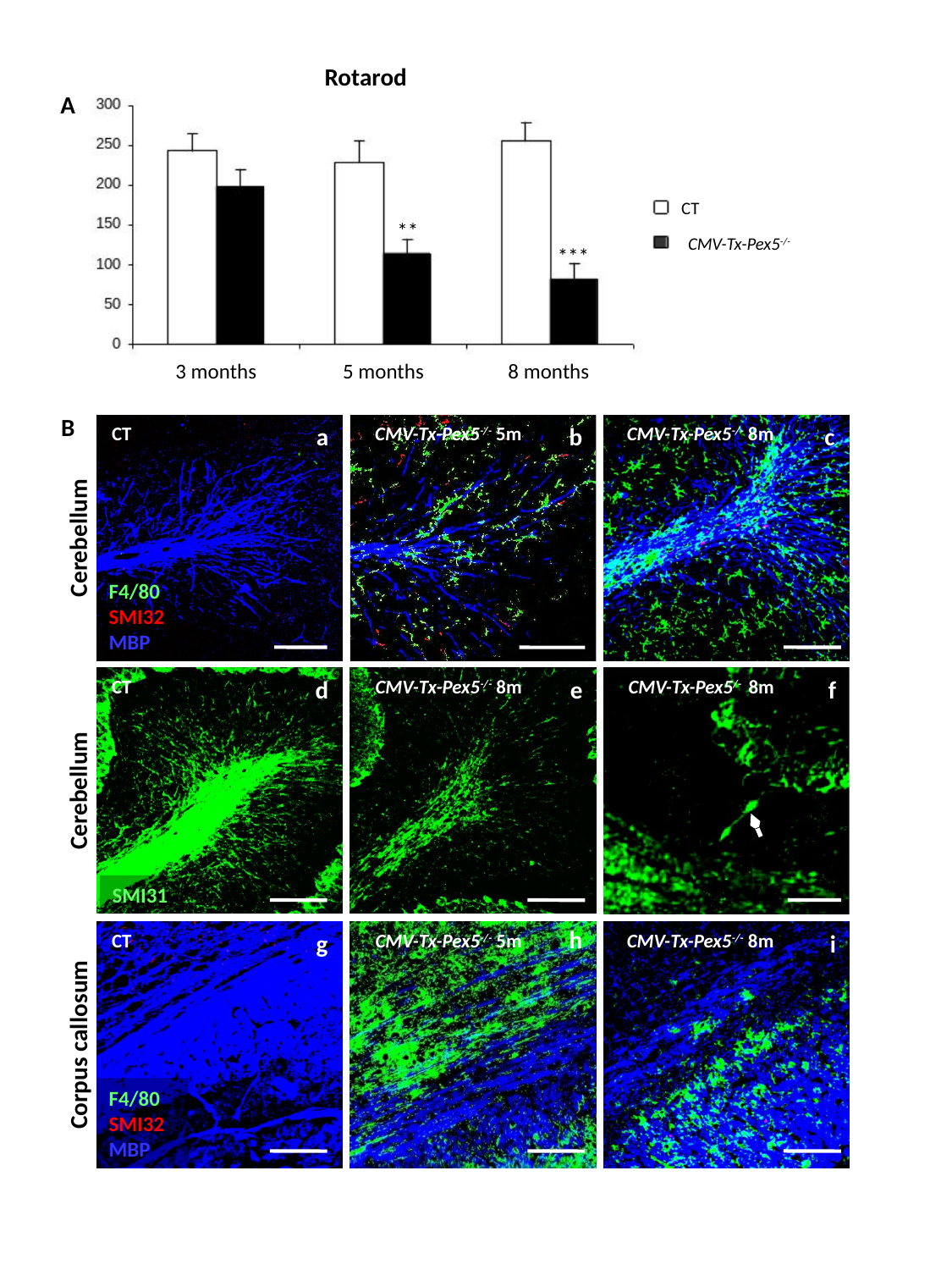

Rotarod
A
CT
**
**
CMV-Tx-Pex5-/-
***
***
3 months
5 months
8 months
B
CT
a
CMV-Tx-Pex5-/- 5m
b
CMV-Tx-Pex5-/- 8m
c
Cerebellum
F4/80
SMI32
MBP
CT
d
CMV-Tx-Pex5-/- 8m
e
CMV-Tx-Pex5/- 8m
f
Cerebellum
SMI31
h
CT
g
CMV-Tx-Pex5-/- 5m
CMV-Tx-Pex5-/- 8m
i
Corpus callosum
F4/80
SMI32
MBP
